# Supplementary material for: Herbarium specimens reveal a constrained seasonal climate niche despite diverged annual climates across a wildflower clade
Source: Proc Natl Acad Sci U S A. 2025 Jul 1;122(28):e2503670122. doi: 10.1073/pnas.2503670122 (PMC12280893; doi:10.1073/pnas.2503670122)
Supplement: Supplementary file 1 — Appendix 01 (PDF) [file pnas.2503670122.sapp.pdf]

Supplemental material for:

Herbarium specimens reveal a constrained seasonal climate niche despite diverged annual climates across a wildflower clade

Megan Bontrager[1,2,3,] Samantha J. Worthy[1,4], N. Ivalú Cacho[5], Laura Leventhal[1,6,7,8], Julin N. Maloof[9], Jennifer R. Gremer[1,2] Johanna Schmitt[1,2], Sharon Y. Strauss*[1,2]

*Corresponding author: systrauss@ucdavis.edu

[1]Department of Evolution and Ecology, UC Davis, Davis, CA, 95616

[2]Center for Population Biology, UC Davis, Davis, CA, 95616

[3]Department of Ecology and Evolutionary Biology, University of Toronto, Toronto, Ontario, Canada, M5S 3B2

[4]School of Biological Sciences, University of Nebraska, Lincoln, NE, 68520

[5]Instituto de Biología, Universidad Nacional Autónoma de México, Ciudad de México, México

[6]Department of Plant Biology, Carnegie Institution for Science, Stanford, CA, 94305

[7]Department of Biology, Stanford University, Stanford, CA, 94305

[8]Department of Integrative Biology, University of California Berkeley, Berkeley, CA, 94720

[9]Department of Plant Biology, UC Davis, Davis, CA, USA, 95616

Supplementary Figures and Tables

Figure S1. Distributions of specimen elevations (m) for each species. Heights of ridges reflect densities of elevation data scaled within each species. Species are ordered based on the phylogeny plotted in Figure 1.

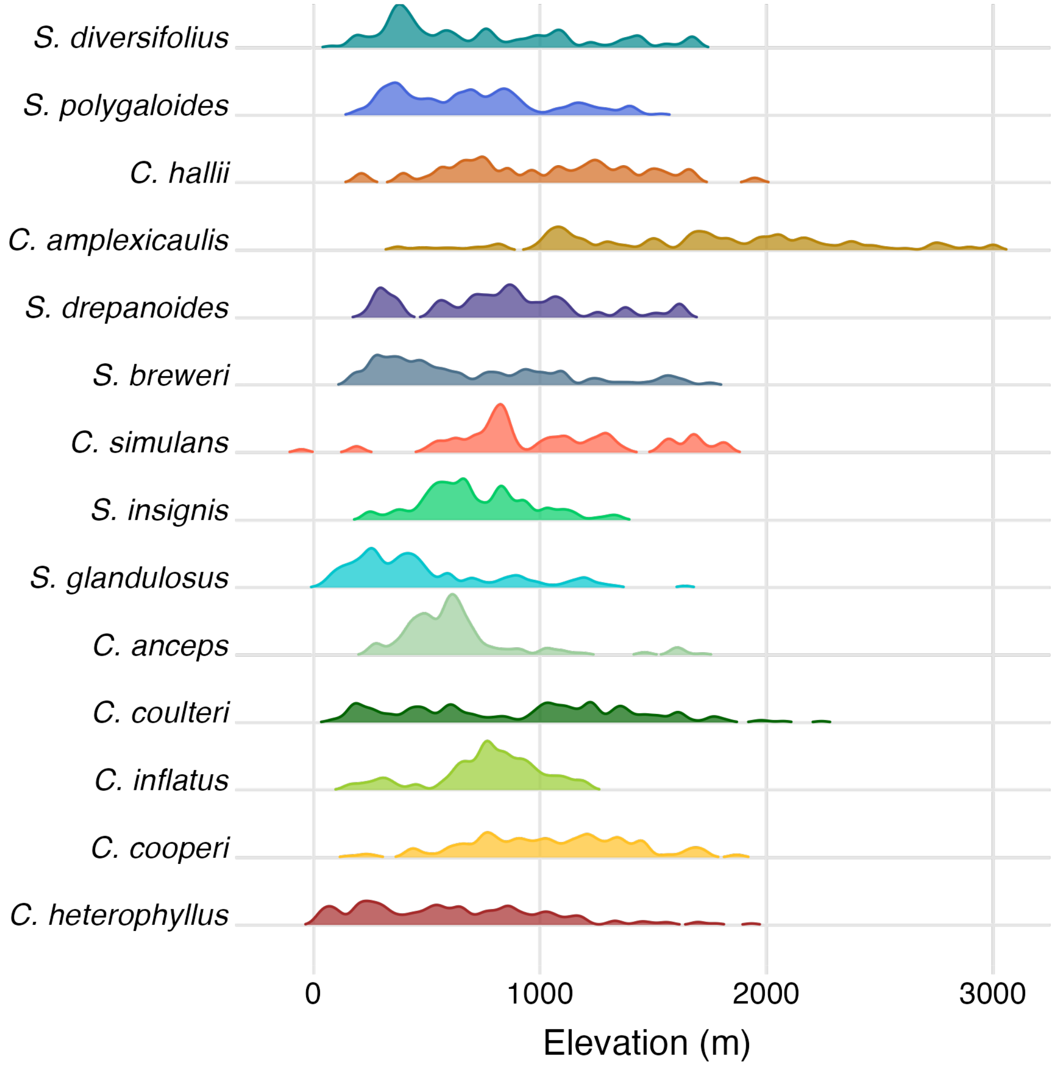

Figure S2. Differences in growing season among the species arranged by latitude (these data are also shown in Figure 1B). Shaded tiles represent the relative proportion of specimens that were estimated to have germinated (blue) or that were collected (red) in a given month for each species. Start month of the growing season was estimated for each specimen as the first month in the fall prior to collection, starting in September, with greater than 25 mm of precipitation. End month of the growing season was the month of collection of each specimen. Specimen-level values were then averaged for species-level estimates of start month and end month, these are depicted with the darker point ranges. *C. amplexicaulis* grows at higher elevations than other species (Figure S1), which may contribute to its deviation from a latitudinal trend in growing season length.

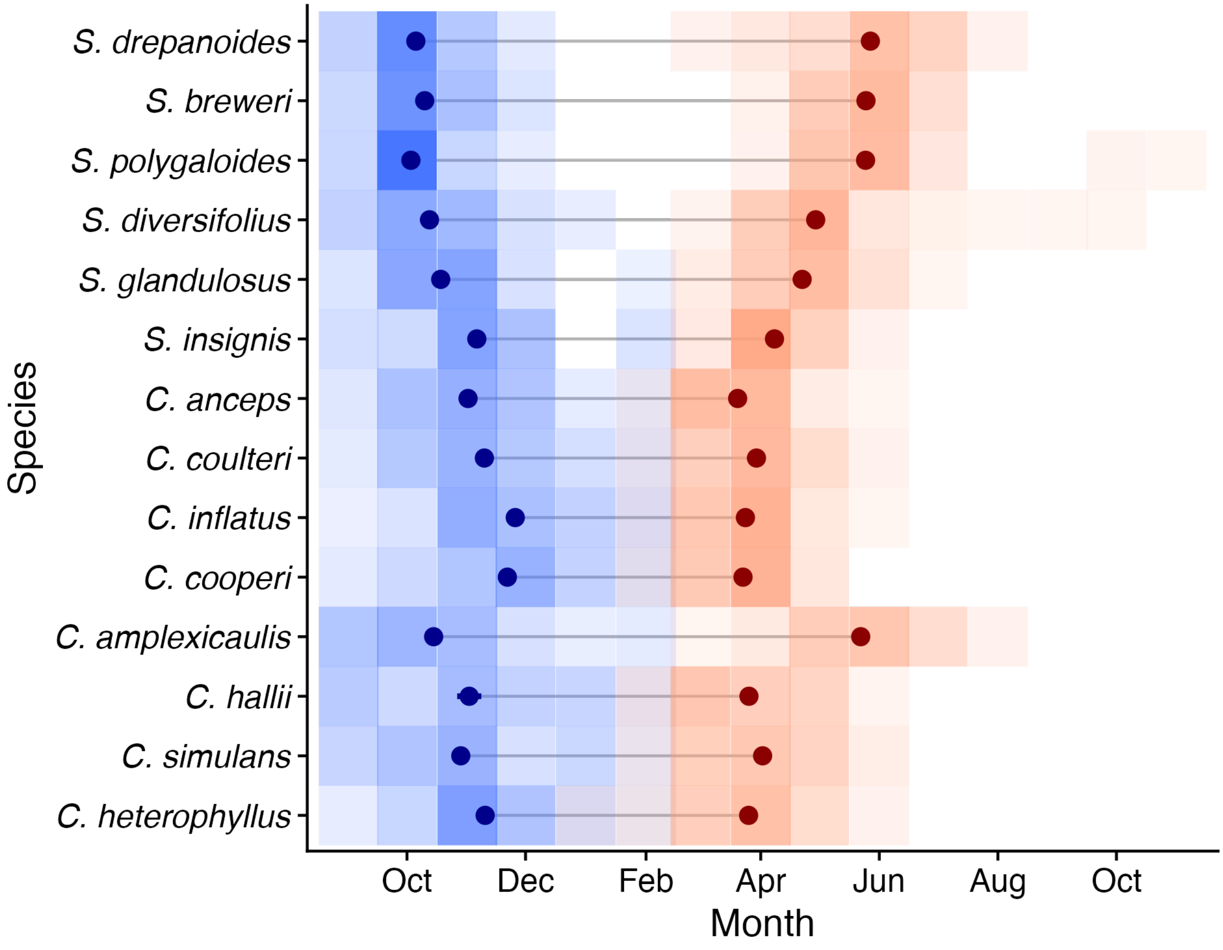

Figure S3. Phenological metrics plotted against latitude. In each panel, trends for individual species are shown with colored lines and the clade-wide pattern is shown with the thick black line. (A.) Species at high latitudes tend to get a first large rain event earlier in the year than those at lower latitudes. (B.) Species at high latitudes tend to be collected later in the year than those at lower latitudes. (C.) Species at high latitudes tend to have longer estimated lifespans than those at low latitudes. Note that trend lines are from linear regressions that do not consider phylogeny and are meant as visual aids rather than statistical estimates.

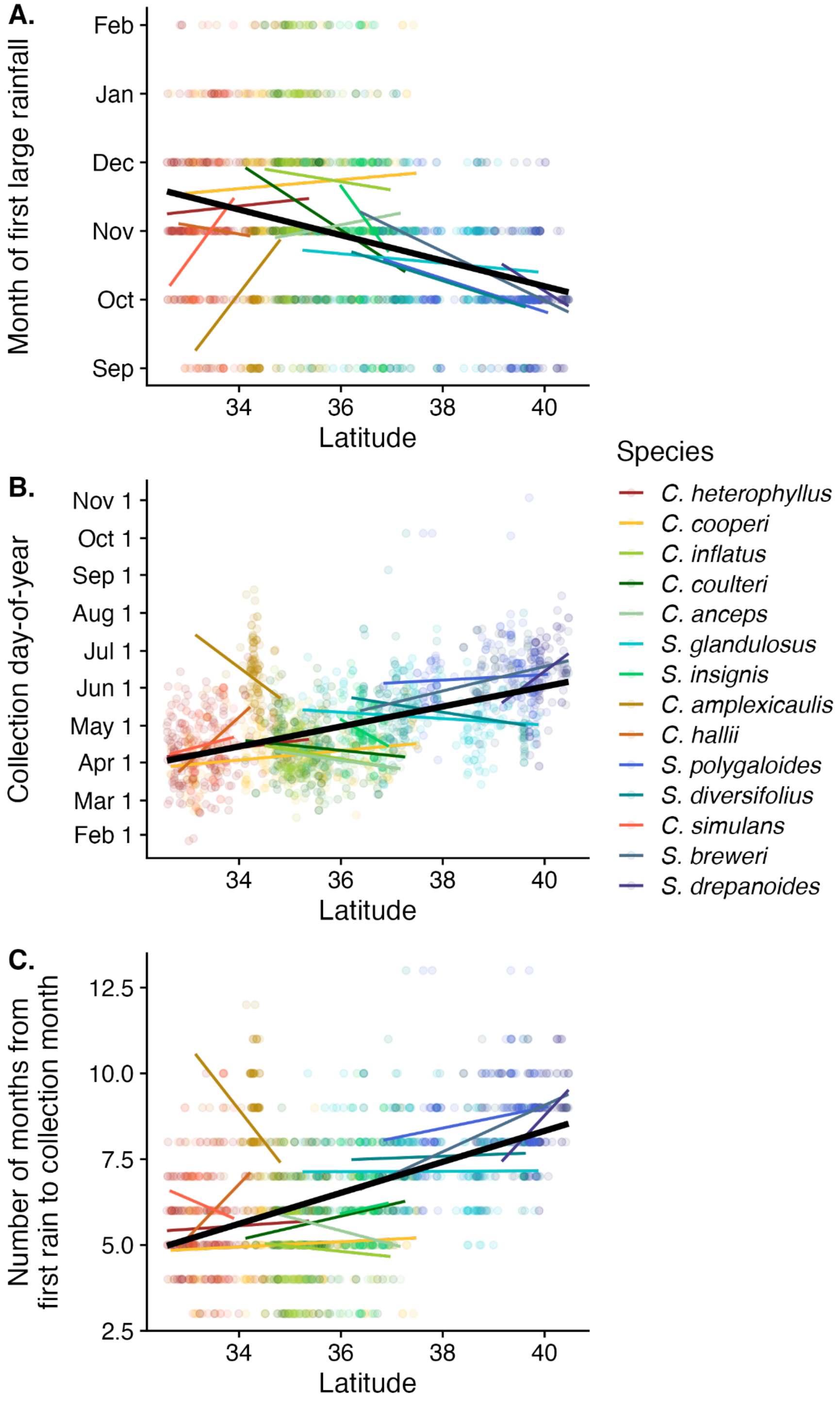

Figure S4. (A.) Climate water deficit (CWD)—which estimates water stress from precipitation, temperature, aspect and soil properties—for annual, average clade-wide seasonal, and specimen-specific seasonal time windows plotted alongside the phylogeny. The same color scale is used for all three time windows. (B.) An alternate visualization of the same annual, average clade-wide seasonal, and specimen-specific seasonal CWD; this panel highlights the variation among specimens within species and the magnitude of differences between the three time windows. Species are in the same order as branch tips in (A.)

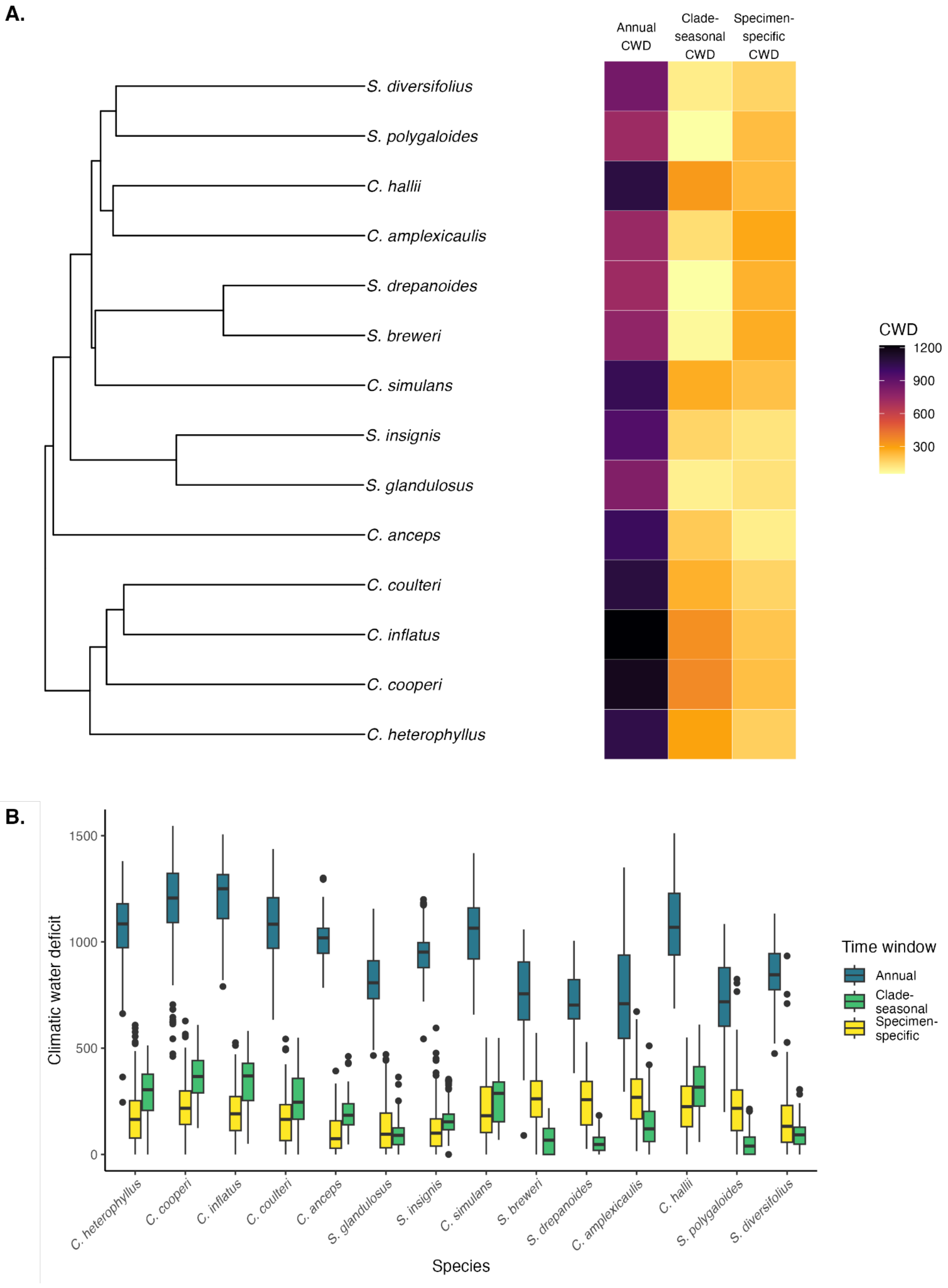

Figure S5. (A.) Average temperature (°C) for the annual, average clade-wide seasonal, and specimen-specific seasonal time windows displayed alongside the phylogeny. The same color scale is used for all time windows to display the differences in average temperature. (B.) An alternate visualization of the same annual, average clade-wide seasonal, and specimen-specific seasonal temperature; this panel highlights the variation among specimens within species and the magnitude of differences between the three time windows. Species are in the same order as branch tips in (A.).

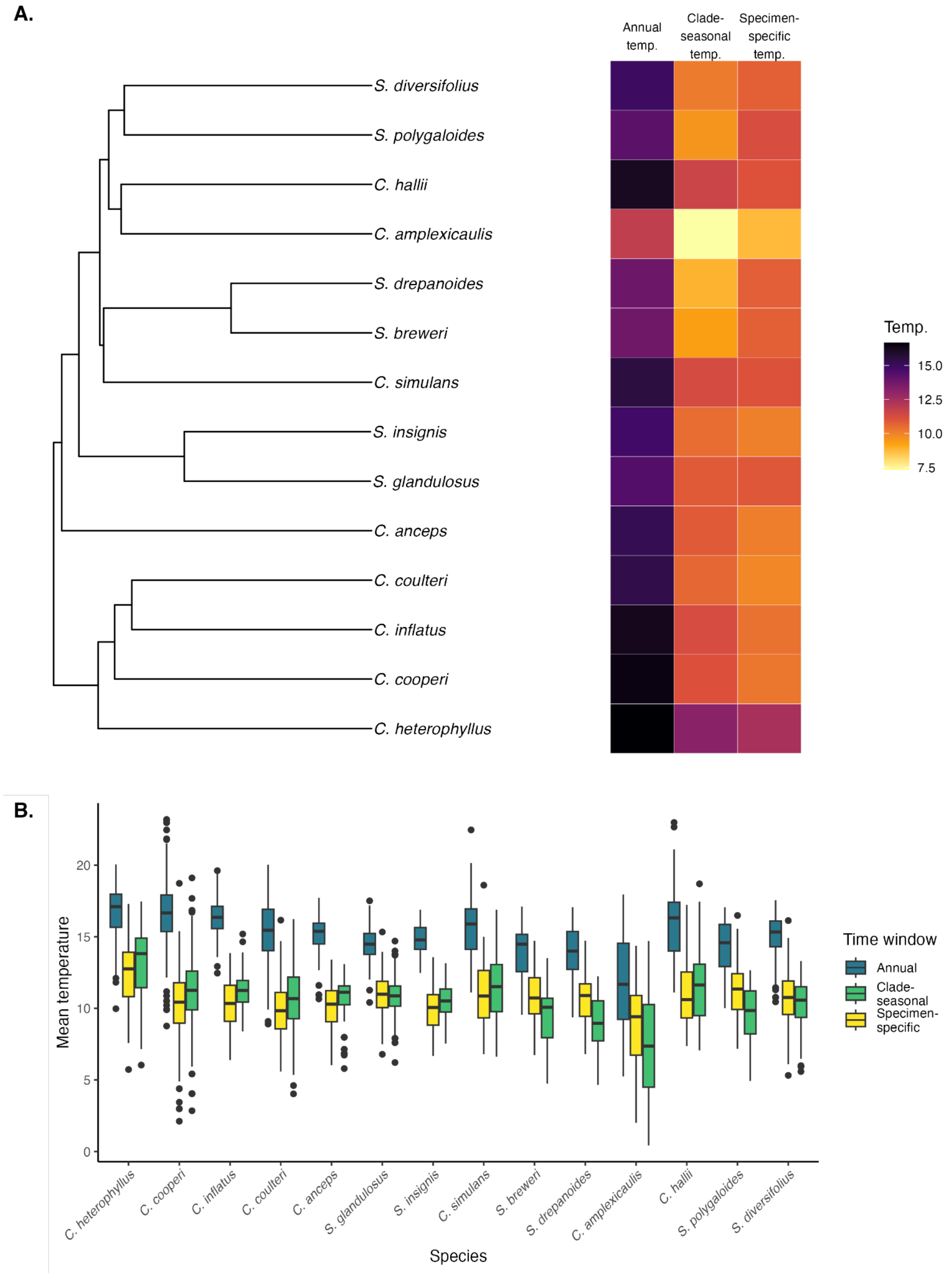

Figure S6. (A.) Summed precipitation (mm) for the annual, average clade-wide seasonal, and specimen-specific seasonal time windows displayed alongside the phylogeny. The same color scale is used for all time windows. (B.) An alternate visualization of the same annual, average clade-wide seasonal, and specimen-specific seasonal precipitation; this panel highlights the variation among specimens within species and the magnitude of differences between the three time windows. Species are in the same order as branch tips in (A.).

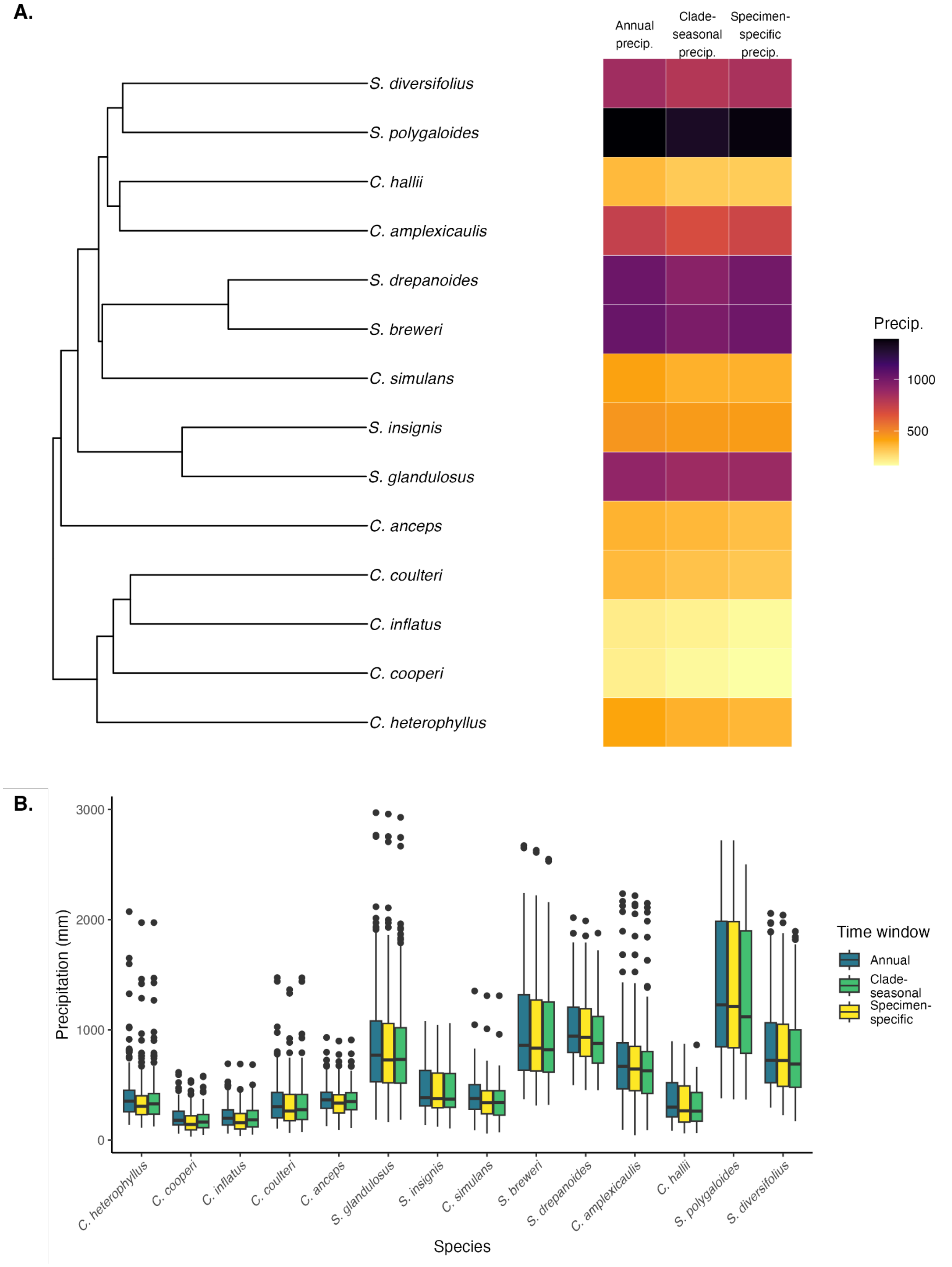

Figure S7. Scatterplots showing relationships over different time scales of average temperature (A.-C.), summed precipitation (D.-E.), and summed climatic water deficit (CWD; G.-I.). Panels are arranged to match the correlation tests in Table 1. (A., D., G.) depict annual vs. clade-seasonal niches. (B., E., H.) depict annual vs. specimen specific niches. (C., F., I.) depict clade-seasonal vs. specimen-specific niches. Correlations are significant in all panels except (H.) and (I.) (see Table 1).

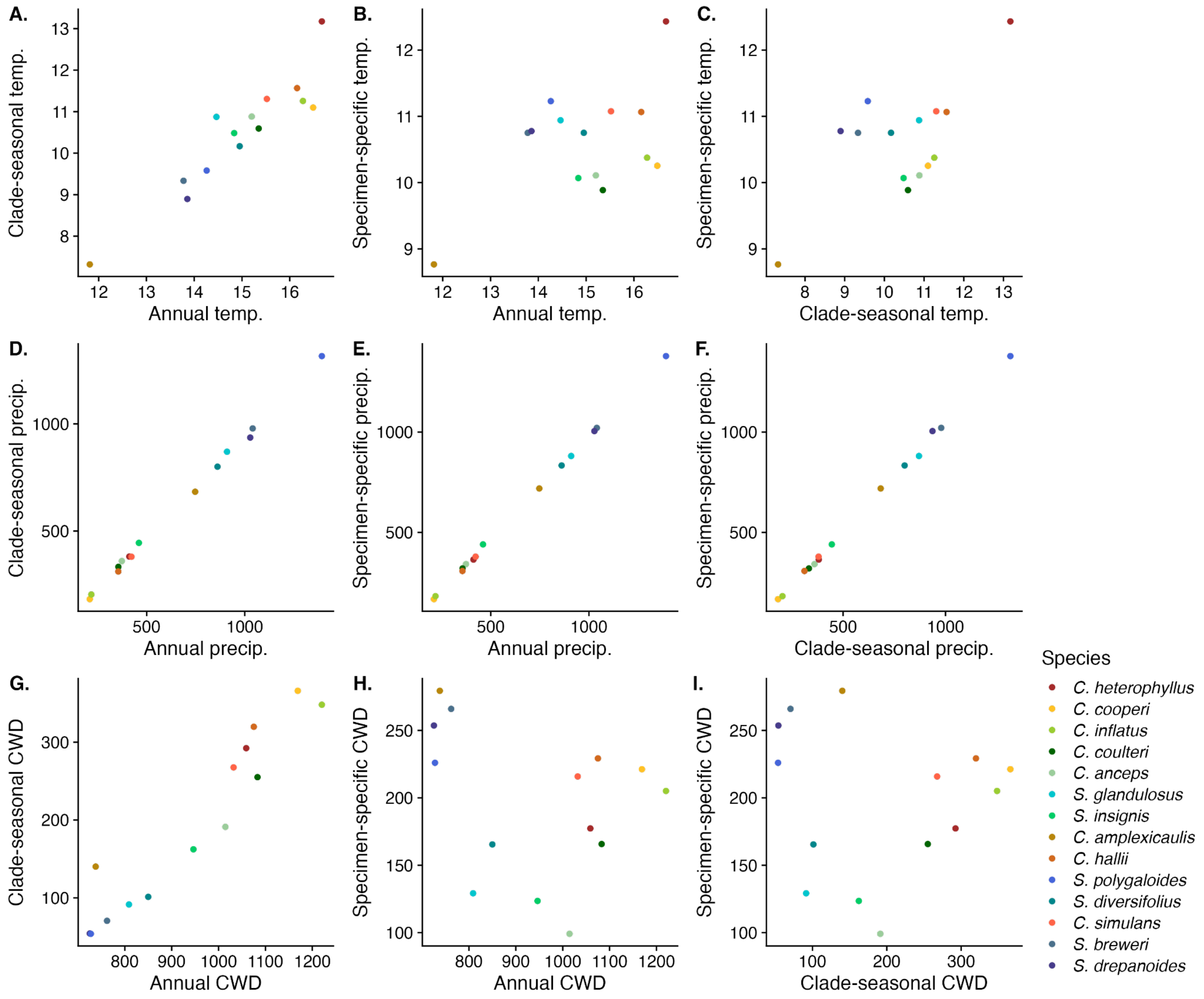

Figure S8. Water holding capacity from occurrence points and from 100 randomly placed points within 20 km of each occurrence. Values were averaged for each species (i.e., averaged across all occurrences for a species, and averaged across the 100 random values x the number of occurrences for each species). Error bars represent standard deviations. Species-averages from occurrence points have lower water holding capacity than the surrounding areas (13/14 species; sign test p = 0.0018).

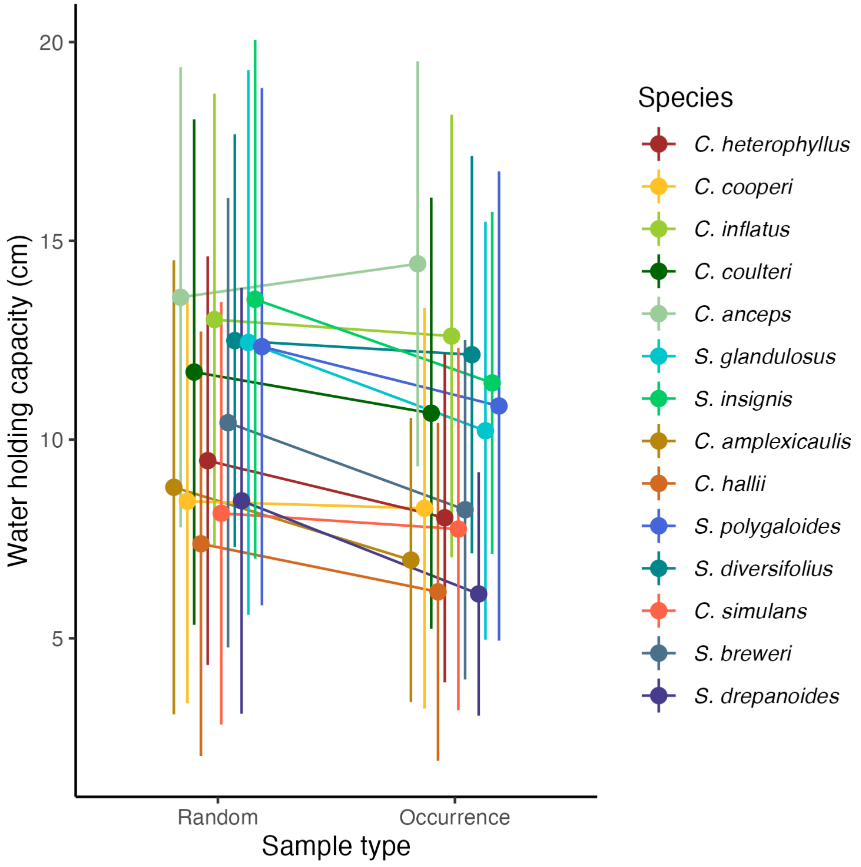

Figure S9. (A.) USDA-NCSS soil survey data within 800 m grid cells illustrating spatial variation in water holding capacity (Walkinshaw et al. 2023). Symbols represent locations of specimens used in this study. (B.) Zoomed in view of the area in the grey square in (A.) showing how specimen occurrences in northern parts of the range are in grid cells with lower water holding capacity than the surrounding areas. Note that color scales differ between the two panels.

A.

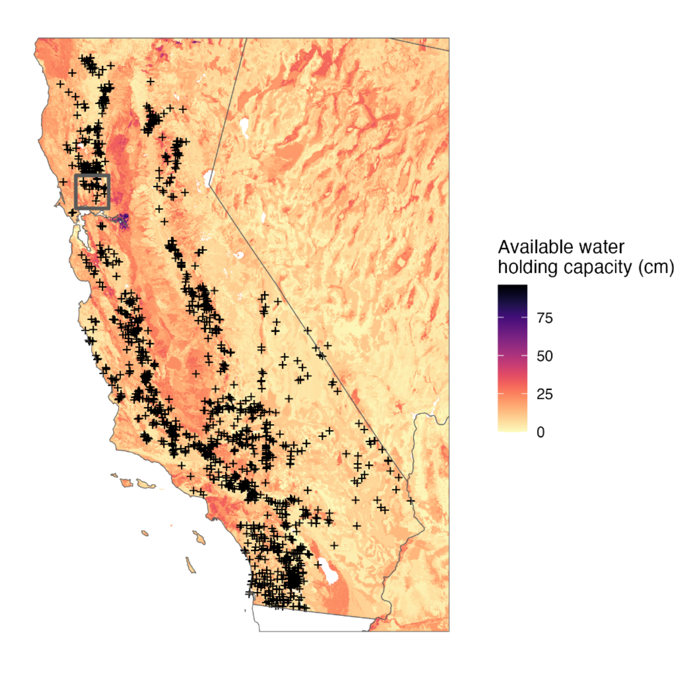

B.

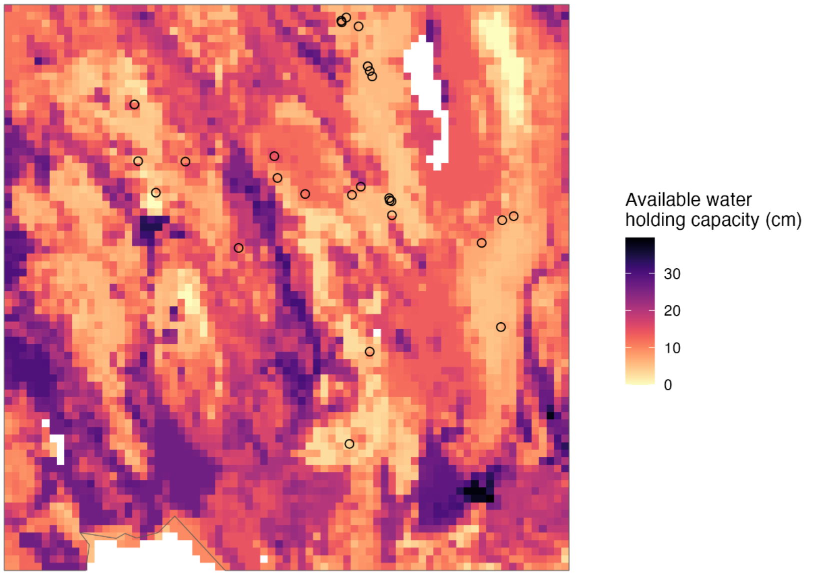

Table S1. Evaluation of phylogenetic signal (Blomberg's K), for each of the climate variables for each time window. The three climate variables were total precipitation (mm), average temperature (°C), and total climate water deficit (CWD). Climate values used in these analyses were species' averages of data from each specimen. When K > 1, p_{bm} refers to the significance of the test for whether there is phylogenetic signal consistent with a Brownian motion model of evolutionary change through time (significant if $p_{bm} < 0.05$). When K > 1 and $p_{bm} <0.05$, we tested whether K significantly differed from 1; p_{gt1} represents a test for whether K is significantly greater than 1, which indicates that trait evolution is more constrained than expected under Brownian motion (significant if $p_{gt1} < 0.05$). Note that there is no phylogenetic signal in the specimen-specific temperature niche, in contrast to other climate variables.

	Annual climate niche	Average clade-wide seasonal climate niche	Specimen-specific seasonal climate niche
Total precipitation	K = 1.22<br>**p_{bm} = 0.0099**<br>p_{gt1} = 0.062	K = 1.20<br>**p_{bm} = 0.013**<br>p_{gt1} = 0.074	K = 1.22<br>**p_{bm} = 0.010**<br>p_{gt1} = 0.055
Average temperature	K = 1.16<br>**p_{bm} = 0.017**<br>p_{gt1} = 0.11	K = 1.12<br>**p_{bm} = 0.034**<br>p_{gt1} = 0.17	K = 0.91<br>p_{bm} = 0.65
Total CWD	K = 1.35<br>**p_{bm} = 0.0029**<br>**p_{gt1} = 0.017**	K = 1.30,<br>**p_{bm} = 0.0052,**<br>**p_{gt1} = 0.026**	K = 1.22<br>**p_{bm} = 0.013**<br>p_{gt1} = 0.060

Table S2. Results of model selection for climate niche evolution. For each combination of climate niche trait and temporal niche, we compared the fit of three models of evolution: 1) Brownian motion (unbounded BM) where climate niche traits evolved as neutral traits as species diverged, 2) Bounded Brownian motion (bounded BM) which is neutral Brownian motion evolution but with bounded constraints, and 3) Ornstein-Uhlenbeck, which is a modified Brownian motion model that constrains climate niche evolution towards a single optimum. Bounded and unbounded Brownian motion models were fit using the phytools package v.1.9.16 (Revell 2024) and the Ornstein-Uhlenbeck model was fit using the geiger package v.2.0.11 (Pennell et al. 2014). The limits for bounded Brownian motion were set as the minimum and maximum values of the climate variables. Model comparisons were made by comparing AIC values. Evolutionary models that were within two units of the lowest AIC model were considered to have similar support. Reported values from the analyses include the step rate, average amount of change expected in each time step (sigsq), the phylogenetic trait mean or value of the root state (x0 or z0), and the log-likelihood of the models (logLik) (Soul and Wright 2021).

Climate variable and time window	Unbounded Brownian motion				Bounded Brownian motion				Ornstein-Uhlenbeck				Best model
	sigsq	x0	logLik	AIC	sigsq	x0	logLik	AIC	sigsq	z0	logLik	AIC	
Temp., annual	988	15.1	-22.2	48.5	1322	15.4	-19.3	46.57	1005	15.2	-22.4	50.7	unbounded and bounded BM
Temp., clade-seasonal	1156	10.7	-23.3	50.7	1190	10.7	-22.0	52.03	1169	10.7	-23.4	52.8	unbounded and bounded BM
Temp., specimen-specific	492	10.6	-17.4	38.7	500	10.6	-16.0	40.05	499	10.6	-17.4	40.9	unbounded and bounded BM
CWD, annual	14684269	970.3	-89.5	183.0	23095197	971.6	-85.2	178.45	14765112	972.5	-89.5	185.1	bounded BM
CWD, clade-seasonal	6465278	211.5	-83.7	171.5	11183800	206.0	-79.1	166.14	6511236	210.0	-83.8	173.6	bounded BM
CWD, specimen-specific	1587124	187.7	-73.9	151.8	2292991	189.1	-71.3	150.55	1595226	188.1	-74.0	153.9	unbounded and bounded BM
PPT, annual	72873562	570.9	-100.7	205.4	107243717	502.9	-96.9	201.76	73240199	566.9	-100.7	207.5	bounded BM
PPT, clade-seasonal	65485821	528.5	-100.0	203.9	96506256	463.3	-96.3	200.58	66372711	528.6	-100.0	206.1	bounded BM
PPT, specimen-specific	75444728	536.9	-100.9	205.9	111804935	467.3	-97.2	202.38	76628431	533.6	-101.1	208.1	bounded BM

Table S3. Proportion of species' occurrences from GBIF in California and the California Floristic Province as of 5/6/ 2025. CA = California. CFP = California Floristic Province, which includes northern Baja California, Mexico, parts of western Nevada and Southern Oregon (Jepson eFlora; https://ucjeps.berkeley.edu/eflora/geography.html). We used the following filters in the GBIF database: Occurrence status: present, Basis of record: herbarium specimen and human observation; Location: coordinates, Continent: North America. GBIF points were not curated for accuracy. In one case (*C. amplexicaulis*), the localities of two observations were disjunct in Arkansas, likely errors in coordinates. All species had >99% of their observations in the California Floristic Province. *C. cooperi* had the lowest proportion of specimens in CA (0.82). Overall, using California-based records provides a good basis for estimating species' climatic range.

Species	California occurrences GBIF	GBIF total occurrences (not checked for accuracy)	Proportion of obs in CA	Proportion in CFP	Occurrences outside CA
C. inflatus	821	824	0.98	1	3 Nevada
C. cooperi	607	740	0.82	1	100 Nev, 29 AZ, 4 Utah
C. heterophyllus	744	784	0.95	1	40 Baja,CA MX
C. coulteri	697	697	1.00	1	0
C. anceps	309	309	1.00	1	0
S. glandulosus	2421	2593	0.91	1	1 Oregon (no other states with records on map)
S. insignis	169	169	1.00	1	0
C. amplexicaulis	271	273	0.99	0.99	2 Arkansas, probably erroneous
C. hallii	241	241	1.00	1	0
S.polygaloides	336	337	1.00	1	0
S. diversifolius	241	241	1.00	1	0
C. simulans	206	206	1.00	1	0
S. breweri	434	434	1.00	1	0
S. drepanoides	171	171	1.00	1	0

References

1. L. J. Revell, phytools 2.0: an updated R ecosystem for phylogenetic comparative methods and other things). *PeerJ* **12**, e16505 (2024).

2. M. W. Pennell, *et al.*, geiger v2.0: an expanded suite of methods for fitting macroevolutionary models to phylogenetic trees. *Bioinforma. Oxf. Engl.* **30**, 2216–2218 (2014).

3. M. Walkinshaw, A. T. O'Geen, D. E. Beaudette, Soil Properties, California Soil Resource Lab. *Soil Prop. Calif. Soil Resour. Lab* (2023). Available at: https://casoilresource.lawr.ucdavis.edu/soil-properties/ [Accessed 30 January 2025].

4. L. C. Soul, D. F. Wright, Phylogenetic Comparative Methods: A User's Guide for Paleontologists. *Elem. Paleontol.* (2021). https://doi.org/10.1017/9781108894142.
